# Probing the stochastic property of endoreduplication in cell size determination of *Arabidopsis thaliana* leaf epidermal tissue

**DOI:** 10.1371/journal.pone.0185050

**Published:** 2017-09-19

**Authors:** Kensuke Kawade, Hirokazu Tsukaya

**Affiliations:** 1 Okazaki Institute for Integrative Bioscience, Okazaki, Aichi, Japan; 2 National Institute for Basic Biology, Okazaki, Aichi, Japan; 3 Department of Basic Biology, School of Life Science, Graduate University for Advanced Studies (SOKENDAI), Okazaki, Aichi, Japan; 4 Department of Biological Sciences, Graduate School of Science, University of Tokyo, Bunkyo-ku, Tokyo, Japan; University of California Irvine, UNITED STATES

## Abstract

Cell size distribution is highly reproducible, whereas the size of individual cells often varies greatly within a tissue. This is obvious in a population of *Arabidopsis thaliana* leaf epidermal cells, which ranged from 1,000 to 10,000 μm^2^ in size. Endoreduplication is a specialized cell cycle in which nuclear genome size (ploidy) is doubled in the absence of cell division. Although epidermal cells require endoreduplication to enhance cellular expansion, the issue of whether this mechanism is sufficient for explaining cell size distribution remains unclear due to a lack of quantitative understanding linking the occurrence of endoreduplication with cell size diversity. Here, we addressed this question by quantitatively summarizing ploidy profile and cell size distribution using a simple theoretical framework. We first found that endoreduplication dynamics is a Poisson process through cellular maturation. This finding allowed us to construct a mathematical model to predict the time evolution of a ploidy profile with a single rate constant for endoreduplication occurrence in a given time. We reproduced experimentally measured ploidy profile in both wild-type leaf tissue and endoreduplication-related mutants with this analytical solution, further demonstrating the probabilistic property of endoreduplication. We next extended the mathematical model by incorporating the element that cell size is determined according to ploidy level to examine cell size distribution. This analysis revealed that cell size is exponentially enlarged 1.5 times every endoreduplication round. Because this theoretical simulation successfully recapitulated experimentally observed cell size distributions, we concluded that Poissonian endoreduplication dynamics and exponential size-boosting are the sources of the broad cell size distribution in epidermal tissue. More generally, this study contributes to a quantitative understanding whereby stochastic dynamics generate steady-state biological heterogeneity.

## Introduction

Variation in cell size is widely observed among different tissues, as well as within a tissue in multicellular organisms. This poses a fundamental question of how cell size diversity is specified in a developmental context-dependent manner. Cell size regulation has been well studied, with particular focus on the mechanism of maintaining constant size by coordinating cell division and growth [1–5]. However, much less is known about how variation in cell size is reproducibly generated from a single clonal origin. Stochasticity is emerging as an element giving rise to a heterogeneous cellular distribution in multicellular tissues [6–11], similar to the stochastic dynamics of gene expression in single-cell organisms [12–14]. It is thus of great interest to quantitatively link a stochastic cellular property to steady-state variation in cell size within a theoretical framework.

In plant leaves, epidermal pavement cells (EPCs), derived from a surface layer of shoot apical meristem [15–17], exhibit striking variation in size, ranging from 1,000 to 10,000 μm^2^ in projected areas when mature [18,19]. Although there is cell-to-cell communication between EPCs and sub-epidermal palisade mesophyll cells (PMCs) [20–22], these two cellular populations are kept separate to form clonally independent tissues. This means that an isoclonal population achieves a diverse EPC size distribution. The mitotic cell cycle occurs throughout the young leaf primordia, and this activity is then arrested in the distal part along a developmental axis [23–25]. After exiting the mitotic cell cycle, some cells enter an endoreduplication phase, in which the nuclear genome is replicated without cell division [26,27]. Chromosomal copy number (ploidy, C) is consequently doubled when one endoreduplication is completed. Endoreduplication takes place for up to four rounds in EPCs until maturation [28]. There is a tight correlation between ploidy level and EPC size, but not PMC size [18, 26–30], suggesting that endoreduplication may be a core mechanism for producing variation in EPC size. Whereas the molecular components involved in endoreduplication have been identified [26–30], the type of mathematical model applicable to the occurrence of endoreduplication, and the way in which endoreduplication contributes quantitatively to the wide variation in EPC size, remain largely unclear.

Variability in the timing of the exit from the mitotic cell cycle was proposed to be a source of EPC size variation in plant sepals, which, like leaves, contain cells of various sizes [7,10]. The decision-making in this model is stochastic; that is, whether a cell continues the mitotic cell cycle or starts endoreduplication is a random process. Once a cell enters the endoreduplication phase, it repeats again and again, causing a continuous increase in ploidy level throughout cellular maturation. This model intuitively explains that if a cell exits mitotic division earlier in its development (and enters endoreduplication earlier), it will have a longer period for rounds of endoreduplication to increase ploidy, resulting in a larger cell. An important assumption of this model is the incremental adjustment of the probability of entering the endoreduplication phase at every round to reproduce the experimentally measured ploidy profile. Although this model effectively recapitulates ploidy profiles and resultant EPC size distribution in sepals, some modification is might be required for examining EPC size distribution in leaves, because the onset and completion of cellular differentiation seem to successively occur along the developmental axis in leaves [23]. This strongly suggests that the duration of the endoreduplication phase is more uniform in leaf EPCs, in contrast to the previous assumption in sepals. Constructing a theoretical framework for EPC size distribution in the leaf and comparing it with the previous framework for the sepal is important for understanding how cell size variation is generated in a developmental context-dependent manner.

Given these observations, we did not consider the time lag in stopping mitotic division but rather postulated that each cell is subject to an endoreduplication phase of the same duration for simplicity. Our major focus here was the stochastic dynamics of the occurrence of endoreduplication within a population, because this is helpful for estimating a steady-state EPC size distribution. A recent study determined EPC and PMC sizes and corresponding ploidy levels using a novel *in situ* imaging technique [28]. This enables us to directly connect the cell size with the nuclear ploidy cell-by-cell in each epidermal and sub-epidermal palisade mesophyll tissue to study their relationship. Here, we quantitatively analyzed this ploidy profile and identified a statistical rule behind the stochastic dynamics of endoreduplication occurrence. We then constructed a mathematical model to estimate the time evolution of ploidy profiles based on this finding. This analysis further allowed us to artificially generate the EPC size distribution in the leaf. We compared simulation data with experimental observations to test whether our theoretical simulation was plausible *in vivo*. On this basis, we propose a simple but viable summary explanation of how characteristic EPC size variation is generated.

## Results

### Long-tail distribution of EPC size in leaves

**Fig 1:**
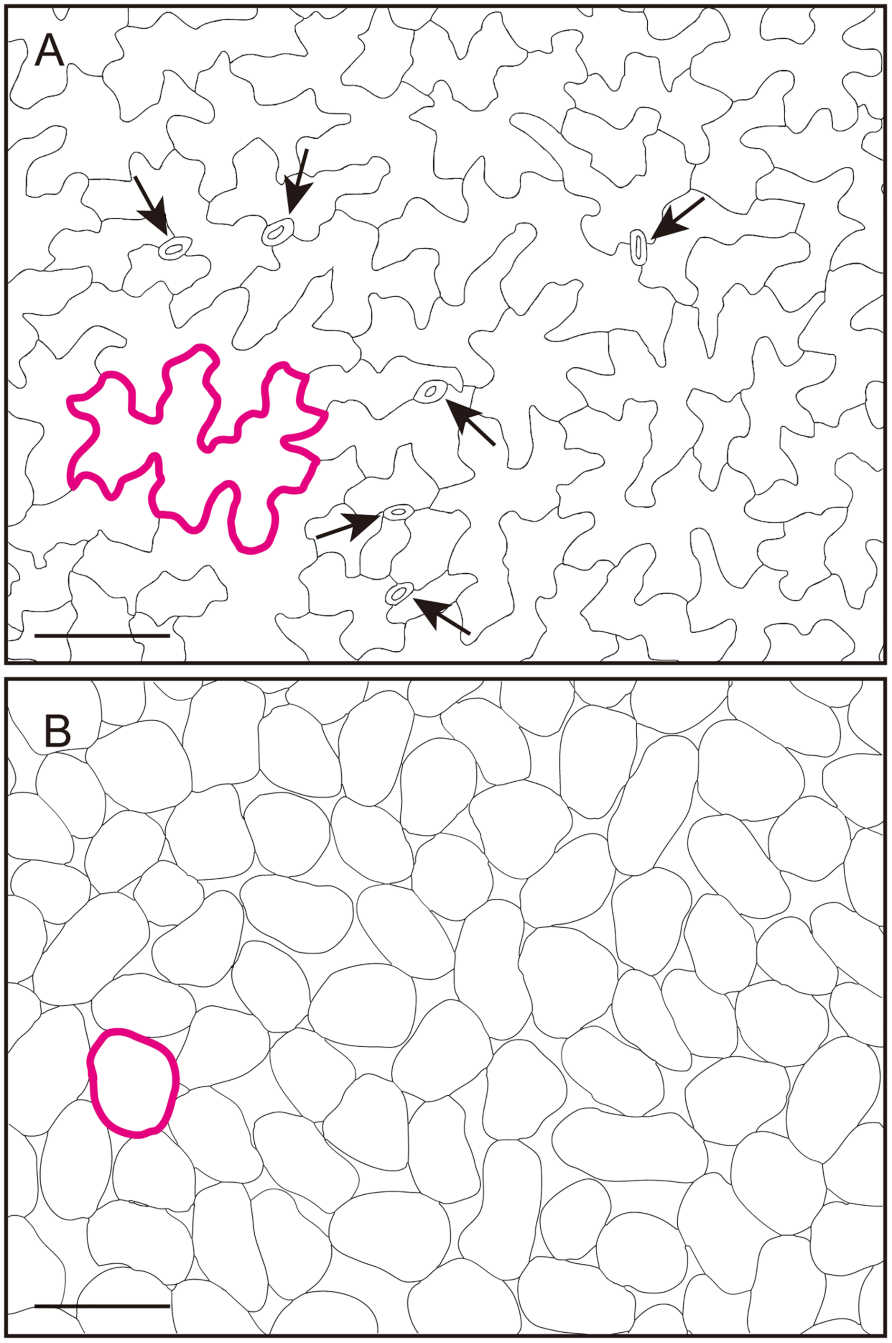
Epidermal pavement and palisade mesophyll cells in Arabidopsis leaf tissue. (A and B) Traced images of adaxial epidermal pavement cells (EPCs) (A) and palisade mesophyll cells (PMCs) (B) in the mature Arabidopsis leaf. Each single cell is outlined in magenta to aid visualization. Arrows indicate stomata guard cells, which are quite different in size as compared with other pavement cells. Scale bars = 100 μm.

EPCs are more variable in size than PMCs in *Arabidopsis thaliana* (Arabidopsis) leaves (Fig 1). In this study, we considered a situation in which EPCs, in contrast to PMCs, have options with regard to their size distribution. More generally, we considered a theoretical framework in which cellular heterogeneity is created from a single origin within a multicellular tissue. This issue is related to various developmental phenomena [31,32]. It is thus important to understand how EPC size is specified and how this cellular trajectory differs from that of PMCs.

**Fig 2:**
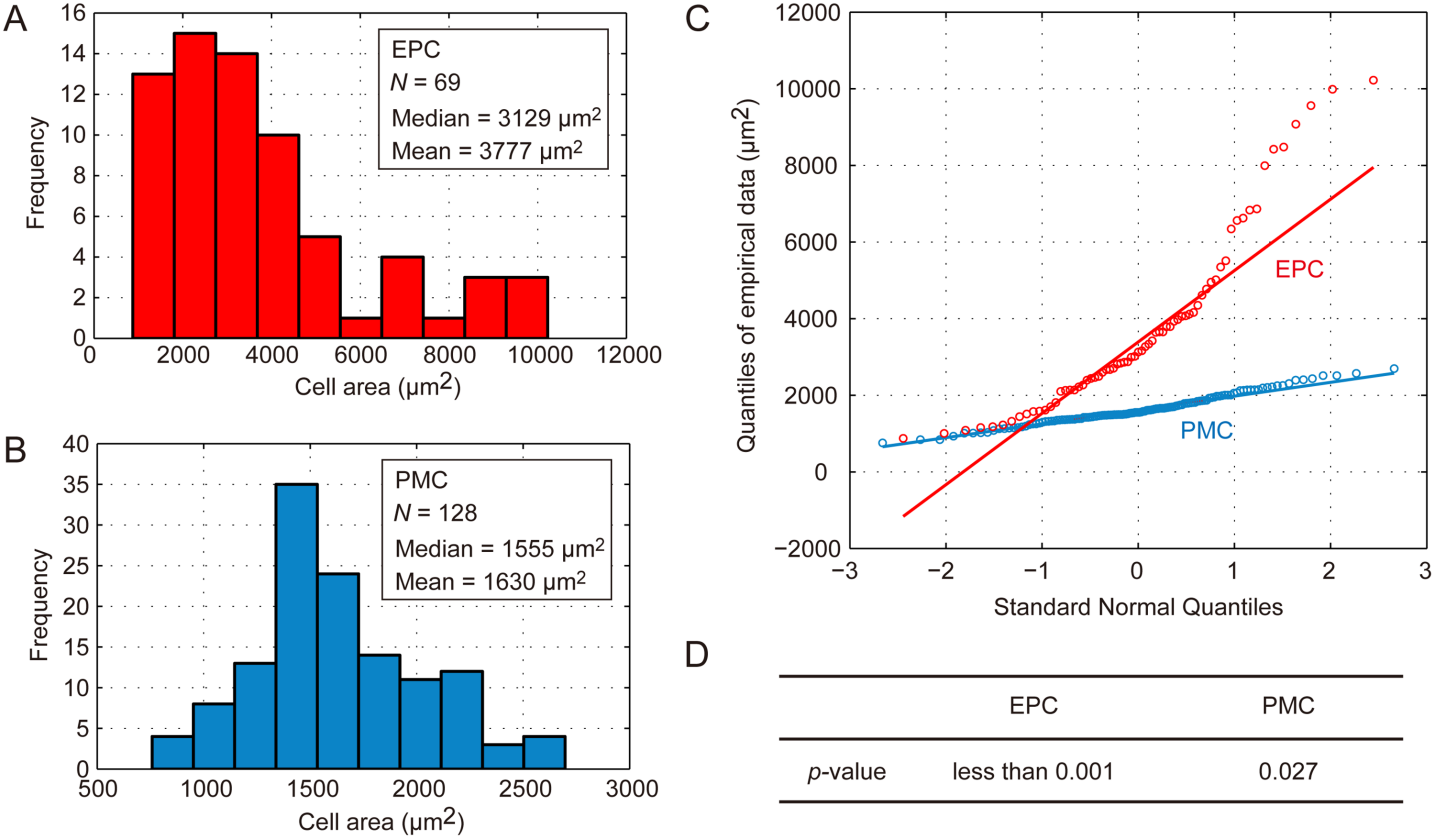
Statistical analysis of cell size distribution in the leaf. (A and B) Histograms of EPC (A) and PMC (B) sizes. Median and mean values of 69 and 128 cells from more than three independent leaf samples for EPCs and PMCs, respectively, are shown. (C) A quantile normal probability plot for size distribution of EPCs (red) and PMCs (blue). Quantiles of the measured cell size data are plotted as a function of theoretical quantiles of the Gaussian estimate. Individual lines aid visualization. (D) The *p*-values from Lilliefors tests for the size distributions of EPCs and PMCs. The null hypothesis assuming normality was rejected with higher confidence for EPCs than PMCs.

We obtained experimental data on EPC and PMC sizes (projected areas) [28] and analyzed the distributions. Whereas PMC size was symmetrically distributed around 1,600 μm^2^ (1,630 ± 391 μm^2^), the EPC size distribution exhibited a longer tail, from the most frequent value of 3,129 μm^2^ to larger cells with a maximum size of 10,226 μm^2^ (Fig 2A and 2B). We plotted quantile values of the EPC and PMC sizes as a function of Gaussian quantiles to statistically dissect the size distributions (Fig 2C). Although the quantile of PMC size formed a roughly straight line corresponding to the Gaussian quantile, EPC size had larger cells than expected from the Gaussian distribution (Fig 2C). This suggests that the Gaussian distribution does not explain the EPC size distribution. Statistical analyses by Lilliefors test also confirmed this result (Fig 2D). These analyses clarified the difference between EPC and PMC sizes and provided a basis for further theoretical study of these distributions.

### Stochastic property of endoreduplication dynamics

**Fig 3:**
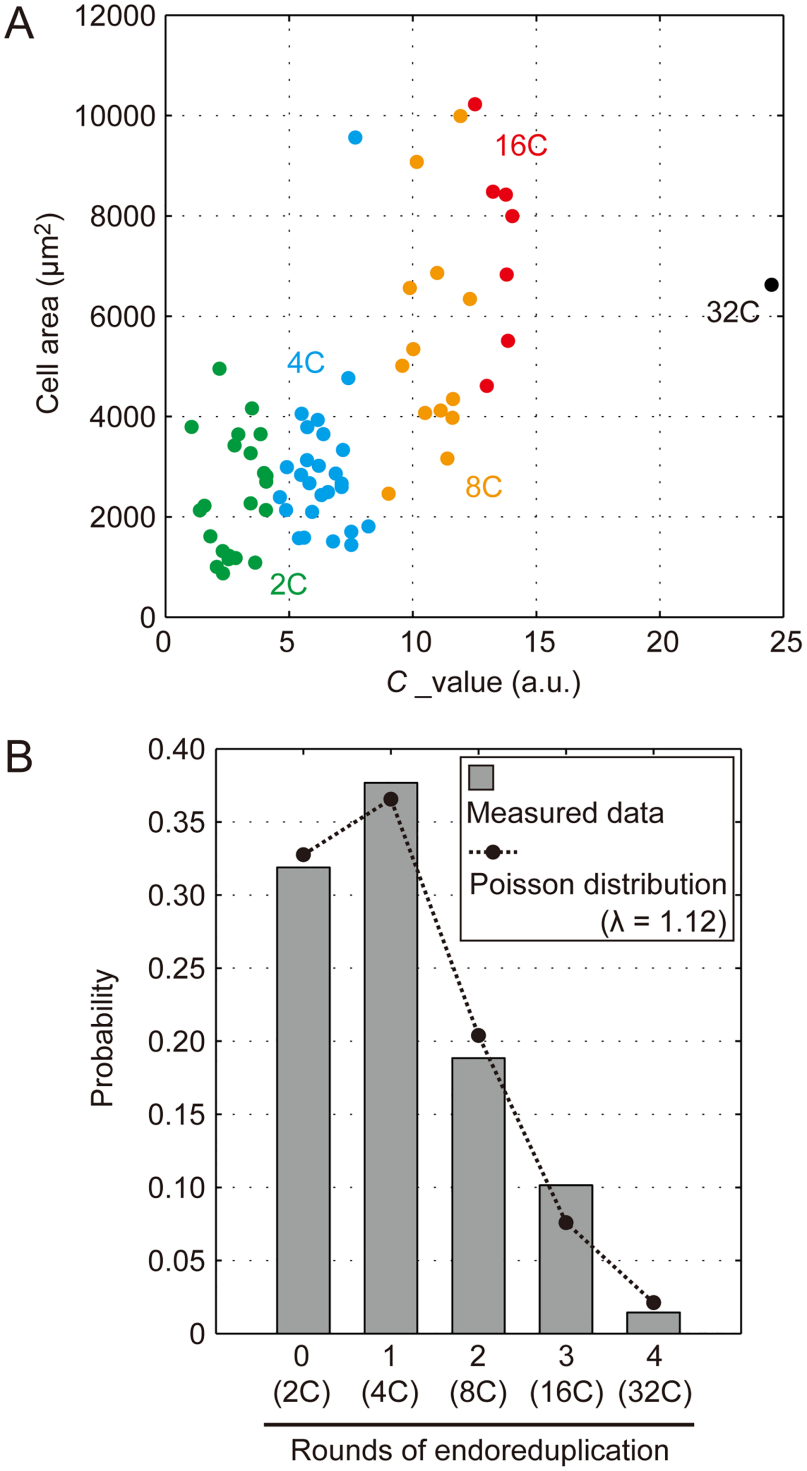
Stochastic property of endoreduplication. (A) The measured size of EPCs plotted against the corresponding ploidy level represented as the *C* value, which indicates DNA content estimated by a 4’,6-diamidino-2-phenylindole (DAPI)-based imaging technique [28]. Data on 2C (green), 4C (blue), 8C (orange), 16C (red), and 32C (black) are shown. This figure is essentially the same as Fig 2C in Katagiri et al., 2016 [28]. (B) Steady-state probability of the occurrence of endoreduplication *in vivo* (gray bars). For example, if endoreduplication occurs four times in a given time period, the ploidy level reaches 32C. The data fit a Poisson distribution (dashed line) with an average of 1.12 rounds.

A series of studies has shown that ploidy level was correlated with EPC size [19,28] (Fig 3A). However, the way in which the occurrence of endoreduplication is determined in a given timeframe remains unclear. To further elucidate this process, we analyzed steady-state ploidy profiles of EPCs using the same dataset used for the cell size analysis [28] (Fig 2A and 2B). According to the data, 4C was the most frequent population (38%), followed by 2C (32%), 8C (19%), 16C (10%), and 32C (1%) [28] (Fig 3B). We found that this profile was well fitted by a Poisson distribution with an average of 1.12 for endoreduplication occurrence per cell (Fig 3B). The ploidy profile estimated by the Poisson distribution was as follows: 2C (33%), 4C (37%), 8C (21%), 16C (8%), and 32C (2%). This indicates that a random process governs endoreduplication dynamics, irrespective of ploidy level, in the leaf epidermis.

### Mathematical model for time evolution of ploidy profile

**Fig 4:**
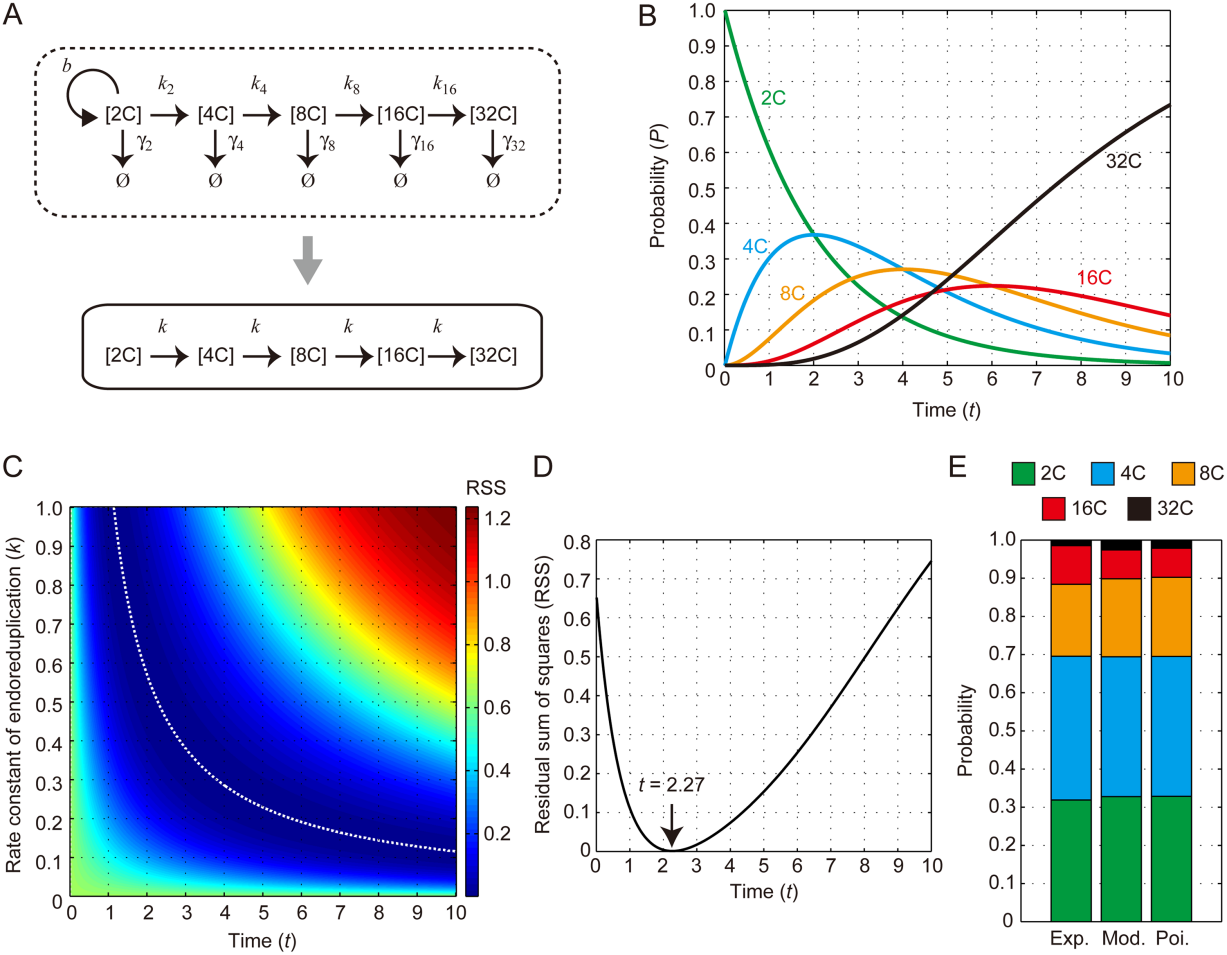
Description of the mathematical model for ploidy profile dynamics. (A) Kinetic scheme describing the sequential reaction of endoreduplication. *k*_2_^*i*^ and *γ*_2_^*i*^ denote the rate constant of endoreduplication from 2^*i*^C to 2^*i*+1^C cells and the removal constant of cells from the 2^*i*^C population, respectively (1 ≤ *i* ≤ 5). *b* indicates the rate constant of mitotic cell division of 2C cells. This scheme could be simplified in this case as given by a single constant rate of endoreduplication *k* in time. (B) Time evolution of the probability of each ploidy obtained by the analytical solution with *k* = 0.5. (C) Parameter dependency for optimizing a ploidy profile with the mathematical model. Color-coded RSS values with various parameter combinations are shown. The white dotted line indicates a minimum RSS in a combination of the parameters *k* and *t*. The color bar is scaled from minimum to maximum in this diagram. (D) Valley-like dynamics of the relationship between *t* and RSS with *k* = 0.5. The RSS reached the minimum when *t* = 2.27. (E) Based on the relationship between *t* and RSS with *k* = 0.5, an optimized ploidy profile was obtained using *t* = 2.27. The probability distribution of each ploidy level from the experimentally measured data (Exp.), the mathematically modeled data at *t* = 2.27 (mod.), and the Poisson distribution (Poi.) are indicated. The probabilities of 2C (green), 4C (blue), 8C (orange), 16C (red), and 32C (black) are shown.

We next examined the time evolution of the ploidy profile by constructing a mathematical model implementing a sequential occurrence of endoreduplication (Fig 4A). Because endoreduplication takes place only after the mitotic cell cycle is exited, the total cell number in the tissue remains constant. This means cell division is negligible, namely, *b* = 0. Cell death and/or cell movement toward the outside of a tissue is undetectable during leaf development, indicating that the removal constant *γ* can be assumed as *γ* = 0. Based on our finding that the occurrence of endoreduplication is a Poisson process irrespective of ploidy level, the rate constant of endoreduplication, *k*_2_^*i*^, was defined as a single constant as:

$$k_{2}=k_{4}=k_{8}=k_{16}=k_{32}=k.$$ (1)

We summarized the model for an increase in ploidy from 2C to 32C by the following differential equations:

$$\frac{dP_{2^{i}}}{dt}=-kP_{2^{i}}\left(i\;=\;1\right)$$ (2)

$$\frac{dP_{2^{i}}}{dt}=k\left(P_{2^{i-1}}-P_{2^{i}}\right)\;\left(i\;=\;2,3,4\right),$$ (3)

$$\frac{dP_{2^{i}}}{dt}=kP_{2^{i-1}}\left(i\;=\;5\right),$$ (4)

where *P*_2_^*i*^ and *t* denote a probability of 2^*i*^C cells and relative time duration between beginning of endoreduplication and measurement time point, respectively (Fig 4A). We set an initial condition of a uniform 2C population, given as:

$$P_{2}\left(0\right)=1,$$ (5)

$$P_{4}\left(0\right)=P_{8}\left(0\right)=P_{16}\left(0\right)=P_{32}\left(0\right)=0.$$ (6)

These differential equations were rigorously solved as follows:

$$P_{2}\left(t\right)=e^{-kt},$$ (7)

$$P_{4}\left(t\right)=P_{2}\left(t\right)kt,$$ (8)

$$P_{8}\left(t\right)=P_{4}\left(t\right)\frac{1}{2}kt,$$ (9)

$$P_{16}\left(t\right)=P_{8}\left(t\right)\frac{1}{3}kt,$$ (10)

$$P_{32}\left(t\right)=1+\;\frac{1}{6}e^{-kt}\left(-6-kt\left(6+kt\left(3+kt\right)\right)\right).$$ (11)

The 2C population decreased exponentially over time. The other populations, with the exception of the 32C population, sequentially increased, and then decreased, as the calculation time proceeded. The 32C population gradually increased and finally became dominant (Fig 4B). These dynamics are known as a gamma distribution [33,34]. We then systematically calculated the residual sum of squares (RSS) of the analytically solved ploidy profile against the measured one, as follows:

$$RSS=\;\sum {\left(expP_{2^{i}}-modP_{2^{i}}\right)}^{2}\;\left(1\leq i\leq 5\right),$$ (12)

where *expP*_2_^*i*^ and *modP*_2_^*i*^ denote the probabilities of experimentally determined and analytically calculated 2^*i*^C cells, respectively. This analysis showed that *t* is uniquely determined to optimize a ploidy profile if we set an arbitrary *k* value in this model and vice versa (Fig 4C). This is intuitively clear because the analytical solution was determined by a combination of two parameters, *t* and *k*. For example, when we performed a numerical simulation with our model using *k* = 0.5 and plotted a relationship between the RSS and *t*, the result showed a valley-like shape (Fig 4D). The RSS achieved a minimum at *t* = 2.27 (Fig 4D). Importantly, the mathematical model successfully reproduced the measured ploidy profiles (Fig 4E), indicating that this is a highly plausible model for explaining ploidy profiles *in vivo*.

### Theoretical simulation for predicting size distribution of EPCs based on ploidy profile

The positive correlation between ploidy and cell size in epidermal tissues has been well established [19, 26–28]. We directly calculated the effect of increased ploidy on cell-size distribution (ploidy effect, *α*) based on experimental observations in Fig 3A [28]. The ratio of the mean projected cell area between 2C and 4C (4C/2C) was 1.22, that between 4C and 8C (8C/4C) was 1.85, and that between 8C and 16C (16C/8C) was 1.36. The average of these ratios was 1.48, suggesting that we can describe the ploidy effect of 2^*i*^C cells as follows:

$$\alpha_{i}=\;1.5^{i-1}.$$ (13)

**Fig 5:**
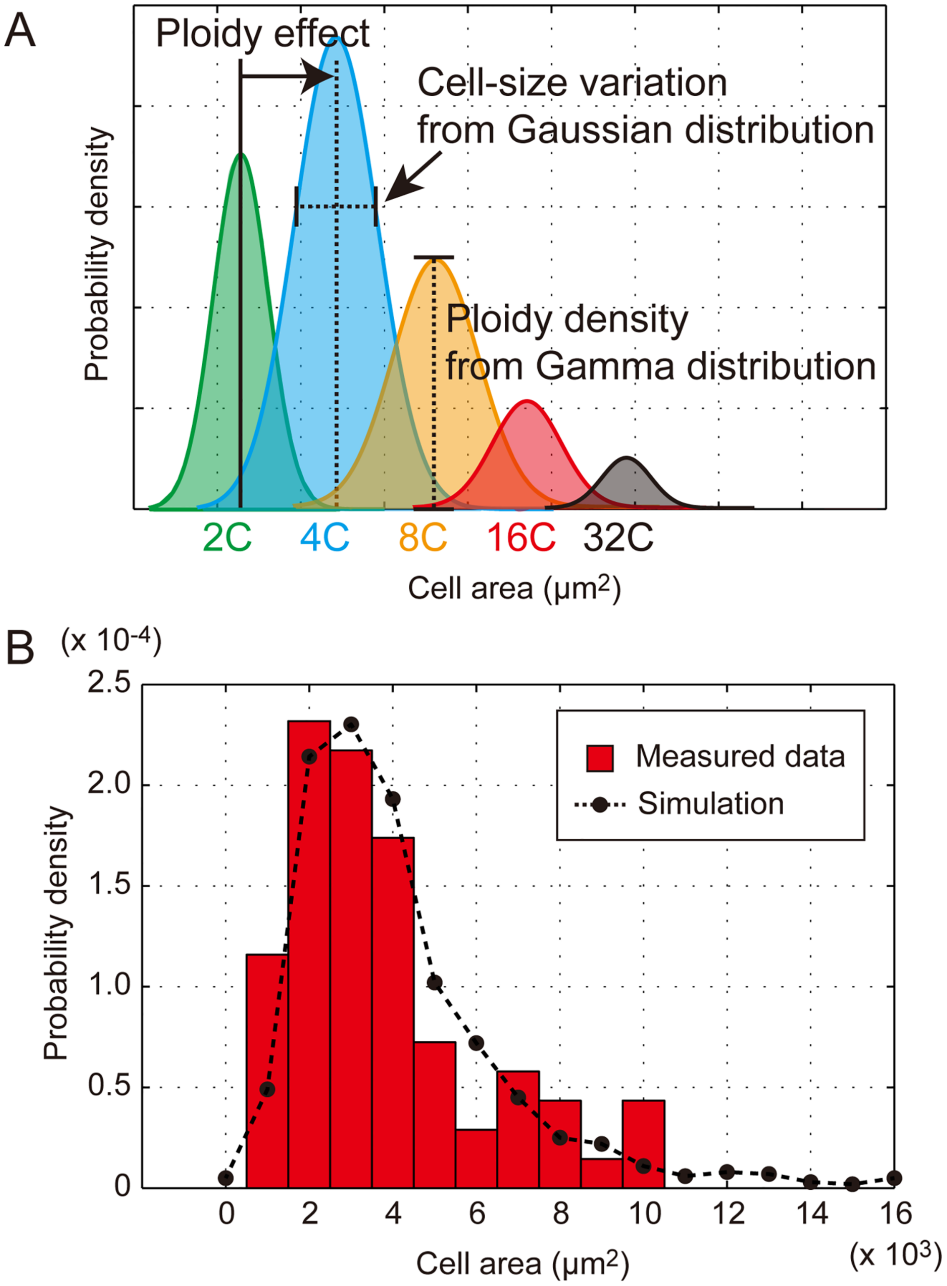
*In silico* test of the theoretical framework for EPC size distribution. (A) Conceptual scheme for determining EPC size distribution. The stochastic property determines the probability density of each ploidy, which makes a steady-state ploidy profile. A Gaussian mean ± standard deviation for cell size is assumed within each ploidy. Because the ploidy effect exponentially enhances cell size, EPC size distributes with a long tail toward larger cells. (B) Histogram of measured EPC size (red bars) and the estimated cell size distribution (black dots with dashed line). The theoretical simulation was performed with our mathematical model using *k* = 0.5 and *t* = 2.27 for predicting the ploidy profile.

This means cell size increases by 50% if ploidy is doubled. Additionally, we assumed that the cell size distribution within each ploidy is derived from the Gaussian distribution. In this scheme, we summarized the mean cell size distribution of 2^*i*^C cells as 2,400*α*_*i*_ μm^2^ and standard deviation as 700*α*_*i*_ μm^2^ in a Gaussian distribution (1 ≤ *i* ≤ 5) (Fig 5A). By incorporating these assumptions into our mathematical model for the ploidy profile, we artificially generated a cell size distribution to investigate whether the EPC size distribution could be attributable to the ploidy profile (Fig 5A). This theoretical simulation predicted a cell size distribution with the most frequent value between 2,500 and 3,500 μm^2^ and a clear long tail toward large cells, which was consistent with experimentally determined data (Fig 5B).

**Fig 6:**
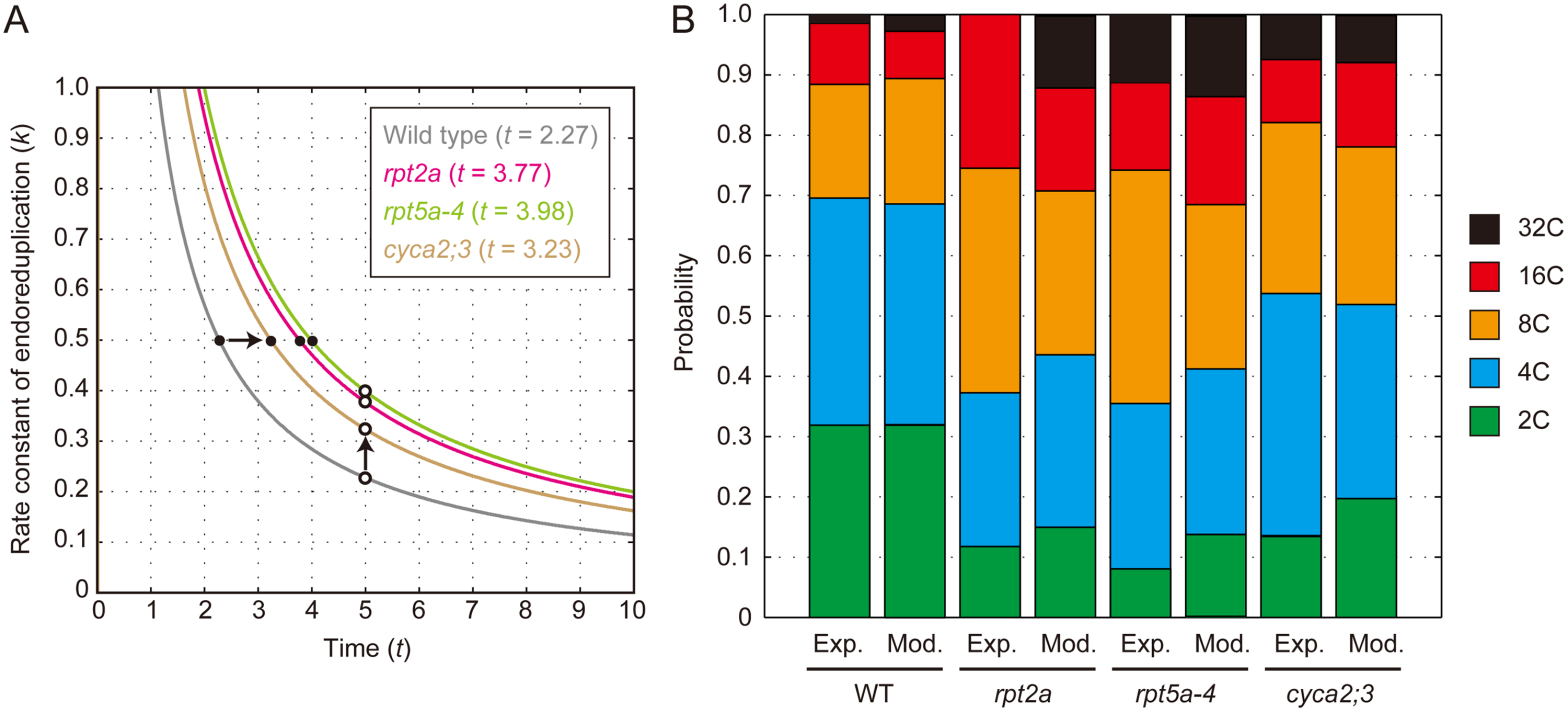
Parameter combinations in endoreduplication-related Arabidopsis mutants. (A) Optimization of the combination of parameters *k* and *t* based on RSS to estimate the ploidy profiles of the *rpt2a*, *rpt5a-4*, and *cyca2;3* mutants. Higher *t* or *k* values were required to minimize RSS if we considered fixed *k* (filled circle) or fixed *t* (open circle) values, respectively, in these mutants. (B) Ploidy profiles determined by mathematical modeling with *k* = 0.5 and the optimized *t* value. Experimentally measured (Exp.) and mathematically modeled (mod.) probabilities of 2C (green), 4C (blue), 8C (orange), 16C (red), and 32C (black) are shown.

To validate the enhancement of cell expansion by the ploidy effect, we analyzed mutants in which the ploidy level was increased. A-type cyclin CYCA2;3 participates in cell-cycle regulation to suppress endoreduplication [35]. RPT2a and RPT5a are molecular components of the ubiquitin/proteasome pathway also involved in negative control of endoreduplication [36,37]. Disruption of each gene extends the endoreduplication phase and/or promotes endoreduplication occurrence, resulting in a higher ploidy profile. We explored a parameter combination that yielded a minimum RSS for these mutants with our mathematical model (Fig 6A). If we set a parameter *k* = 0.5, the ploidy profile could be optimized by a larger *t* value (*t* = 3.77, 3.98, and 3.23 for *rpt2a*, *rpt5a-4*, and *cyca2;3*, respectively) than that for the wild type (WT; *t* = 2.27) (Fig 6A). This suggests that the endoreduplication phase is 1.5–2.0 times more prolonged in these mutants. We then obtained the ploidy profiles of these mutants from our mathematical model. These were consistent with the experimentally determined data, except for an absence of 32C cells in the *rpt2a* mutant (Fig 6B). Differences between measured and simulated probability of each ploidy are less than 0.12 (average = 0.04, Fig 6B). Essentially the same ploidy profile was obtained when we optimized the *k* value with a fixed *t* (Fig 6A and 6B).

**Fig 7:**
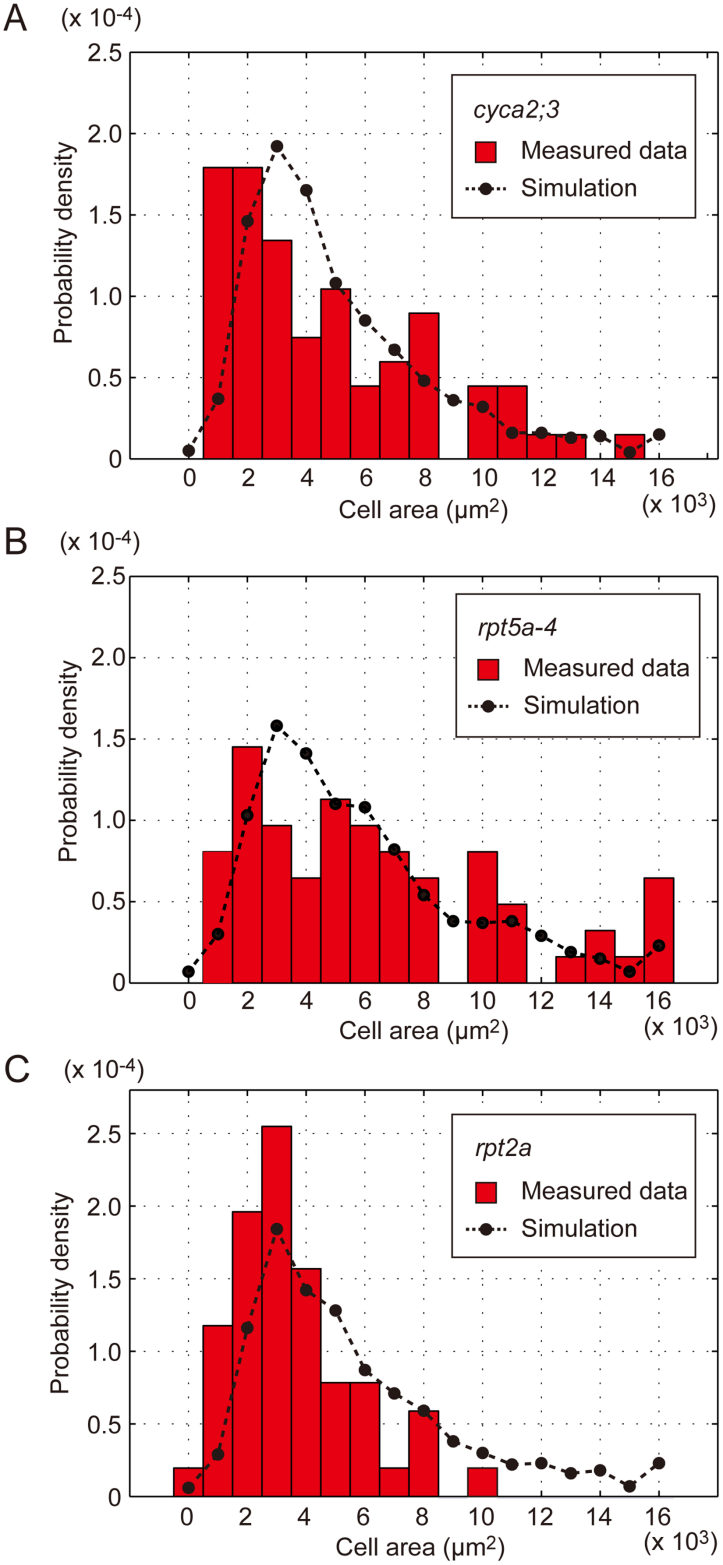
Validation of the ploidy effect using endoreduplication-related Arabidopsis mutants. (A–C) Histograms of the measured size of the EPCs (red bars) and the estimated cell size distribution (black dots with dashed line) in *cyca2;3* (A), *rpt5a-4* (B), and *rpt2a* (C) mutants. The theoretical simulation was performed with *t* = 2.27 (WT), *t* = 3.23 (for *cyca2;3*), *t* = 3.98 (for *rpt5a-4*), and *t* = 3.77 (for *rpt2a*) using a fixed *k* = 0.5 for estimating ploidy profiles.

We observed that the overall cell size distribution was shifted toward large cells ranging from 14,587 μm^2^ in *cyca2;3* to 23,703 μm^2^ in *rpt5a-4 in vivo* (Fig 7A and 7B). Our theoretical simulation for cell size distribution recapitulated this trend with a similar size range (Fig 7A and 7B). This indicates that our theoretical simulation for cell size distribution is viable *in vivo*. Although it has been proposed that RPT2a and RPT5a work in the same pathway [36], cell size distribution in *rpt2a* was similar to that in WT. This is, at least partially, due to the absence of 32C cells in *rpt2a* in the analyzed data. Accordingly, our theoretical simulation did not reproduce this ploidy profile (and resultant cell size distribution). Further investigation of the genetic pathways in which RPT2a and RPT5a are involved and further characterization of the phenotypes of these mutants, in addition to refinement of the theoretical framework, are required to explain this discrepancy between experimental observations and theoretical data.

### Quantitative dissection of the ploidy effect on EPC and PMC sizes

**Fig 8:**
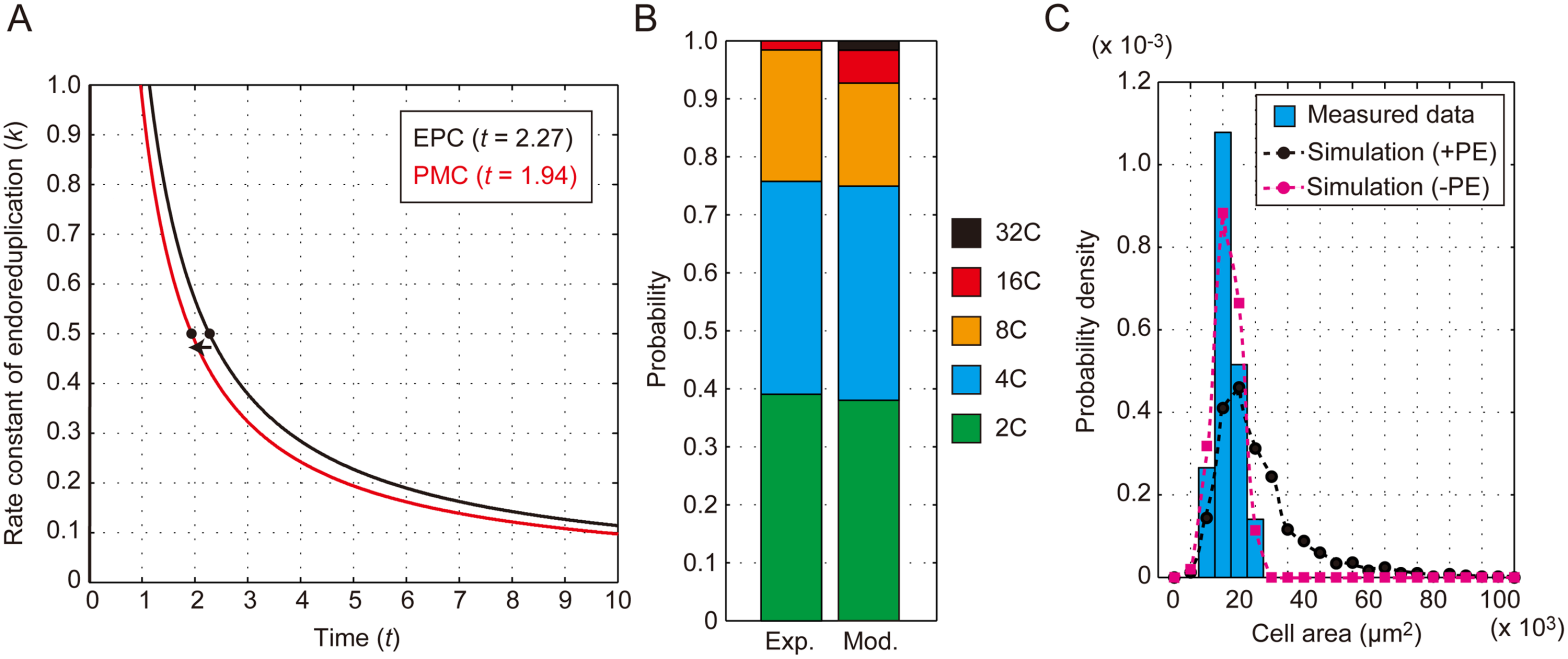
Absence of the ploidy effect in PMC size distribution. (A–C) Optimization of the parameter *t* in our model with *k* = 0.5 based on the RSS value to estimate the ploidy profile of PMCs in WT (A) and the ploidy profile thus determined (B). Experimentally determined (Exp.) and mathematically modeled (mod.) probabilities of 2C (green), 4C (blue), 8C (orange), 16C (red), and 32C (black) are shown. (C) Histogram of the measured size of the PMCs (blue bars) and the estimated cell size distribution with the ploidy effect (+PE, black dots with solid line) or without (-PE, magenta dots with solid line).

Lastly, to identify differences between EPCs and PMCs in terms of cell size determination, we analyzed the ploidy effect on PMC size regulation. We analyzed the parameter combination based on RSS and determined that *t* decreased by 15% in PMCs compared with EPCs when we used a fixed *k* = 0.5 (Fig 8A). This indicates that the endoreduplication phase was slightly shortened (or the occurrence of endoreduplication was slightly suppressed) in PMCs. Although our model predicted the 32C cell population (1.6%) in PMCs, this population is barely detectable *in vivo* [28] (Fig 8B). When we implemented this ploidy effect in our theoretical framework, cell size distribution was still affected and extended toward larger cells (Fig 8C). This was cancelled in the absence of the ploidy effect, resulting in a distribution that was similar to that of the experimentally measured cell size (Fig 8C). We thus concluded that the ploidy effect is a specific element of size regulation in EPCs and has no impact on PMCs.

**Fig 9:**
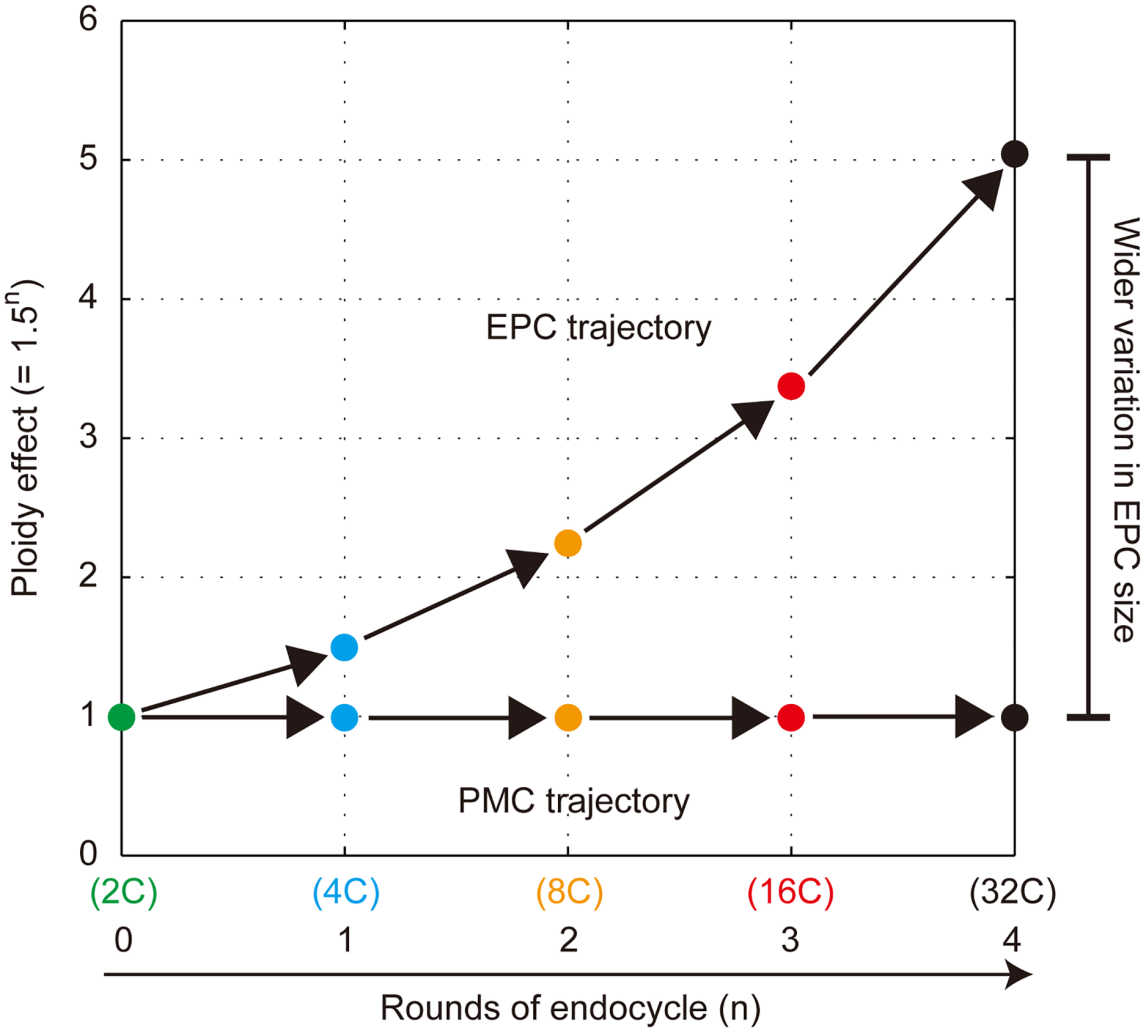
Trajectories of EPC and PMC size determination. Endoreduplication occurs with a single rate constant through the maturation of EPCs and PMCs. The ploidy effect, which increases 1.5 times at every endoreduplication, is evident in the EPCs but not in the PMCs, resulting in a wider variation in EPC size.

In summary, we identified the stochastic property of endoreduplication dynamics as a Poisson process, which allowed us to analytically solve the ploidy profile of EPCs. By incorporating the ploidy effect into our mathematical model, we succeeded in summarizing the trajectory of EPC size determination (Fig 9). This theoretical simulation was sufficient to explain how variation in EPC size is established, except in *rpt2a* mutants. In contrast to the EPCs, endoreduplication was suppressed by 15% and the ploidy effect was hardly detectable in the PMC trajectory (Fig 9), resulting in a rather symmetric cell size distribution. These quantitative and theoretical dissections of the EPC and PMC size distributions clarified a basic strategy of cell size determination in leaves.

## Discussion

Stochasticity often induces a heterogeneous population of cells from a single origin. This is evident not only in bacterial populations but also in cellular communities within tissues [6–14]. It is thus interesting to theoretically link stochastic dynamics with steady-state distributions to elucidate a regulatory design of biological processes. Our study focused on variation in EPC size in Arabidopsis leaves to address this question because of their characteristic cell size distribution. We determined that the endoreduplication dynamics conformed to a stochastic Poisson process, and constructed a theoretical simulation with the ploidy effect to predict EPC size distribution. Because this simulation reproduced the experimentally observed variation in EPC size, we proposed that this is a highly plausible framework for explaining how cell size distribution is established *in vivo*. The most beneficial feature is that our theoretical framework consists of a simple combination of statistical descriptions. This enables us to compare theoretical backgrounds of biological designs to understand developmental phenomena.

### Linking stochastic dynamics of endoreduplication to steady-state cell size distribution

We formulated the endoreduplication progression as a sequential reaction with the rate constant *k* in time *t*. Based on our finding that the occurrence of endoreduplication is a Poisson process, the rate constant between each step could be uniformly defined irrespective of the ploidy level. This means that a balance between these two parameters determined the ploidy profile. Its time evolution was obtained from the mathematical model. Consistent with the analytical solution, the dynamics of each ploidy followed a gamma distribution in time [33,34]. This analysis enabled us to optimize the rate constant *k* at a given time *t* to reproduce the ploidy profile *in vivo*. Our mathematical model thus summarized the progression of endoreduplication in leaves more simply than the previous model in sepals, in which the rate constant was not uniform and was modified at every time point [10]. Based on our model, we provided an intuitive and quantitative explanation for the phenotypes of endoreduplication-related mutants in which the endoreduplication phase was extended (and/or the rate constant of the endoreduplication was increased) by 42% and 75% in the *cyca2;3* and *rpt5a-4* mutants, respectively. This analysis is relevant for quantitatively describing mutant phenotypes associated with endoreduplication disorders; thus, it can be used for designing biological processes.

We incorporated (1) a Gaussian distribution of cell size at each ploidy level and (2) an exponential cell size increase associated with an increase in ploidy in the theoretical simulation to analyze EPC size distribution. This simulation offered a robust fit of experimental data on the distribution in WT and also in the *cyca2;3* and *rpt5a-4* mutants. When we eliminated the ploidy effect from the framework, we recapitulated the PMC size distribution as well. Our recent study revealed that the ploidy effect is tightly associated with epidermal cell identity [28]. We thus concluded that the exponential cell size boost based on stochastic endoreduplication dynamics is a specific source of the long-tailed distribution of EPC size.

### Theoretical background for the induction of heterogeneous populations

We identified a theoretical background for understanding EPC size distribution in leaves. One intensive study on a similarly skewed biological distribution investigated the variation in protein copy number in single bacteria cells [12–14, 38]. In this case, protein copy number was determined by Poissonian transcription of messenger RNA and a subsequent exponential burst of protein translation in a cell. The resultant variation in protein copy number within a bacterial population formed a gamma distribution. Although the details of EPC size and protein copy number distributions differ, it is interesting to note that these steady-state profiles are achieved by exponential boosts upon accumulation of stochastic events. This may be a preferable strategy for producing a heterogeneous population from a single origin in biological systems, posing the question of how cell size is exponentially enlarged by the ploidy effect. One possible explanation is the ploidy-dependent production of a signaling molecule that promotes cell enlargement. However, production of the signaling molecule is not always doubled in association with genome duplication [39,40]. Experimental verification of the hypothetical molecule is essential to address this issue.

### Other possible elements involved in cell size determination

Given the different modes of cellular coin-toss for entering endoreduplication between the leaf and the sepal [7,10,41], we constructed a novel model for predicting ploidy profiles in the leaf. One of the characteristics of sepal EPCs is a giant cell, which stretches 200–400 μm (30 μm in width) along the length of the sepal (around 1,500 μm). In contrast to our model, the previous model predicted giant cells at a realistic rate [7,10]. We thus consider that both models are viable *in vivo* and should be selected depending on the tissue analyzed.

Although we offer a simple theoretical framework to explain cell size distribution, we can consider more complex models involving mechanical feedback and biochemical fluctuation, in addition to a time lag in stopping mitotic division, variation of mitotic cell size and noise of the ploidy effect [8, 23–25, 31, 42,43]. Given that we successfully reproduced ploidy profiles and cell size distributions, it is possible that these elements have already included in our model as intrinsic and/or extrinsic effects. If so, it remains to be determined how these elements were incorporated into our framework. For example, whereas we here assumed that mean and standard deviation of cell-size distribution within each ploidy are proportional with the ploidy effect, this relation is not fully resolved. Detail theoretical and experimental investigations of the ploidy effect on cell-size distribution might be a key to address these issues, as performed in studies of gene expression [44,45]. Therefore, continuous assessment using these elements may be helpful in further refining the framework. To this end, quantitative and theoretical analyses of cell size distributions under situations in which these elements are perturbed are essential for experimentally verifying our proposed framework

## Materials and methods

### Experimentally measured data and statistical analyses

Data on the EPC and PMC projected areas and corresponding nuclear ploidy in the first leaves of 30-day-old plants [28] were used. These data were statistically analyzed with MATLAB_R2012b (MathWorks, Natick, MA, USA).

### Mathematical modeling for ploidy profiling

We considered a sequential reaction for formulating endoreduplication with the rate constant *k* in time *t*. All cells were 2C under the initial conditions. We then calculated the time evolution of the ploidy profile using Eqs 7–11 until the total time reached *t* = 10.00 with a time interval *t* = 0.01, at which point the 32C population was clearly dominant. The ploidy profile was uniformly determined with optimized parameter combinations of *t* and *k* by minimizing the RSS between simulated and experimentally determined profiles using Eq 12. We used Mathematica software (Wolfram, Champaign, IL, USA) to solve the differential equations.

### Theoretical framework for cell size distribution

The EPC size distribution of 1,000 cells was predicted subsequent to mathematical modeling the ploidy profile. We constructed a theoretical simulation based on a Gaussian cell size distribution within each ploidy level. The main assumption in this simulation was the ploidy effect (*α*), which enlarged the projected cell area according to the ploidy level in an exponential manner, as described in Eq 13. By incorporating these elements into our mathematical model for the ploidy profile, we were able to perform a theoretical simulation to obtain the steady-state cell size distribution with a custom-made MATLAB script, which is available upon request.

## Abbreviations

Arabidopsis: *Arabidopsis thaliana*
EPC: epidermal pavement cell
PMC: palisade mesophyll cell
RSS: residual sum of square
WT: wild type
